# Surgical management of spinal metastases originating from thyroid cancer

**DOI:** 10.1186/s12957-026-04371-x

**Published:** 2026-04-29

**Authors:** Majid Esmaeilzadeh, Harold F. Hounchonou, Jörg Andreas Müller, Frank Bengel, Joachim K. Krauss

**Affiliations:** 1 Department of Neurosurgery, Hannover Medical School, Carl-Neuberg-Str. 1, Hannover, 30625 Germany; 2 Department of Neurosurgery, University Hospital OWL, Campus Bielefeld-Bethel, Bielefeld, 33617 Germany; 3 Department of Nuclear Medicine, Hannover Medical School, Carl-Neuberg-Str. 1, Hannover, 30625 Germany

**Keywords:** Thyroid carcinoma, Follicular thyroid carcinoma, Spinal metastases, Spinal cord compression

## Abstract

**Background:**

The spine is an uncommon metastatic location from thyroid cancer. Here, we describe our experience with spinal cord compression as presentation of metastatic thyroid carcinoma, including surgical management and outcome.

**Methods:**

Five patients with spinal metastases from thyroid cancer were identified over a 20-year period.

**Results:**

This descriptive case series comprised 5 women with a median age of 61 years. Three patients presented to the emergency room without a previous diagnosis of thyroid carcinoma. Clinical symptoms at presentation included pain, ataxia, and bladder and bowel incontinence. Imaging (MRI in four patients and CT in one) revealed thoracic spinal metastases in four cases and a sacral lesion in one case. Surgical treatment consisted of en-bloc resection in one patient and subtotal resection in the remaining four. The median Karnofsky Performance Score improved from 70% to 90%, postoperatively. Histopathological analysis confirmed follicular thyroid carcinoma in all cases. Postoperatively, all patients received radioactive iodine therapy, and three patients additionally underwent radiotherapy. One patient had a recurrence. The median survival time was 69 months (range 19–188 months).

**Conclusion:**

The main goals of surgical management in patients with spinal metastases from thyroid cancer are preservation of neurological function and restoration of spinal stability. This is followed by comprehensive evaluation and treatment of the primary malignancy. Multidisciplinary management is essential, with subsequent therapy directed toward control of systemic disease.

## Introduction

Thyroid carcinoma (TC) typically manifests as a slow-growing tumor and is generally associated with a relatively favorable long-term prognosis [1, 2]. The most common subtype of differentiated thyroid TC is papillary thyroid carcinoma (PTC), followed by follicular thyroid carcinoma (FTC) [3, 4]. PTC usually spreads locally through the lymphatic system, while FTC is more likely to disseminate hematogenously, leading to distant metastases in organs such as lungs, brain, and skeleton with spinal metastases occurring in about 3% of patients [5, 6].

Despite the overall favorable prognosis of TC, patients with spinal metastases may experience a diminished quality of life due to pain or neurological complications [7, 8]. Managing spinal metastasis from TC presents a therapeutic challenge and may require surgical interventions to alleviate symptoms [3, 9].

In previous studies, spinal metastases secondary to TC were analyzed in the frame of larger cohorts with various primary tumors or bone metastases [7, 10–13]. Overall, there is a notable lack of research regarding spinal metastases from TC making the management of these patients challenging.

In this study, we detail the clinical presentation, therapeutic approaches, and long-term outcomes of five patients who presented with spinal cord compression secondary to metastatic TC.

## Materials and methods

We performed a retrospective review of a database from the Department of Neurosurgery at Hannover Medical School spanning a 20-year period, identifying patients with spinal metastases originating from TC. The patients’ charts were reviewed to gather demographic and clinical data, including age, sex, symptoms, treatment protocols, pre- and postoperative Karnofsky Performance Scores (KPS), imaging findings, concomitant diseases, histopathological findings, and survival data. The KPS was determined based on pre- and postoperative neurological examinations.

The study was conducted in accordance with the principles of the Declaration of Helsinki; at the authors’ institution, no formal approval from the ethics committee was required for this type of retrospective analysis. All patient data were fully anonymized prior to analysis in accordance with institutional policies and applicable data protection regulations.

## Results

### Cohort description

Between 2005 and 2025, five consecutive patients underwent surgery for spinal metastases from TC in our department. All five patients were included in the final cohort. The demographical and clinical data of patients at the time of diagnosis of the spinal metastases are summarized in Table 1. The median age at spinal metastases diagnosis was 61 years, ranging from 28 to 79 years. All patients were female. The median preoperative KPS was 70%, ranging from 60% to 100%.

**Table 1:** Patients´ characteristics and treatment of spinal metastases

Case	Sex/age (yr)	Location	Clinical presentation	Modality of diagnosis	Surgical procedure	Pathology	Adjuvant therapy
#1	F/55	T11	Pain	MRI and CT	En-bloc resection and dorsal transpedicular fixation	Metastasis from FTC	RAI
#2	F/66	T6	Neuropatic pain	MRI	Subtotal resection and dorsal transpedicular fixation	Metastasis from FTC	RAI + RTx
#3	F/28	Sacrum	Bladder and bowel incontinence	CT	Subtotal resection and sacral reconstruction with a mesh implant	Metastasis from FTC	RAI
#4	F/79	T7	Ataxia	MRI	Subtotal resection	Metastasis from FTC	RAI + RTx
#5	F/77	T3/4	Ataxia	MRI	Subtotal resection	Metastasis from FTC	RAI + RTx

*FTC* follicular thyroid carcinoma, *RAI* radioactive iodine therapy, *RTx* Radiotherapy

### Clinical presentation, medical history and imaging findings

Three patients (cases #2, #3, and #5) presented to the emergency room without a known history of TC or spinal metastases, but with neurological symptoms attributable to spinal involvement, including neuropathic pain, bowel and urinary incontinence and gait ataxia. Two patients (cases #1 and #4) were referred after spinal lesions were detected elsewhere.

Patient #1 had been diagnosed with TC 14 years earlier and was primarily treated with thyroidectomy and lymphadenectomy. During follow-up, she developed multiple bone metastases, including involvement of the cervical, thoracic, lumbar, and sacral spine, which had been managed primarily with radioiodine therapy and, selectively, with radiotherapy. The lesion prompting referral to our department had been identified one year earlier on routine bone scintigraphy and showed progression despite repeated courses of radioiodine therapy. Patient #4 had experienced unexplained dyspnea for 3 months and finally underwent PET-CT, which revealed multiple lesions in the spine, lungs, skull, and stomach. After biopsy of the gastric lesion, she was referred to our department for further management.

**Fig. 1:**
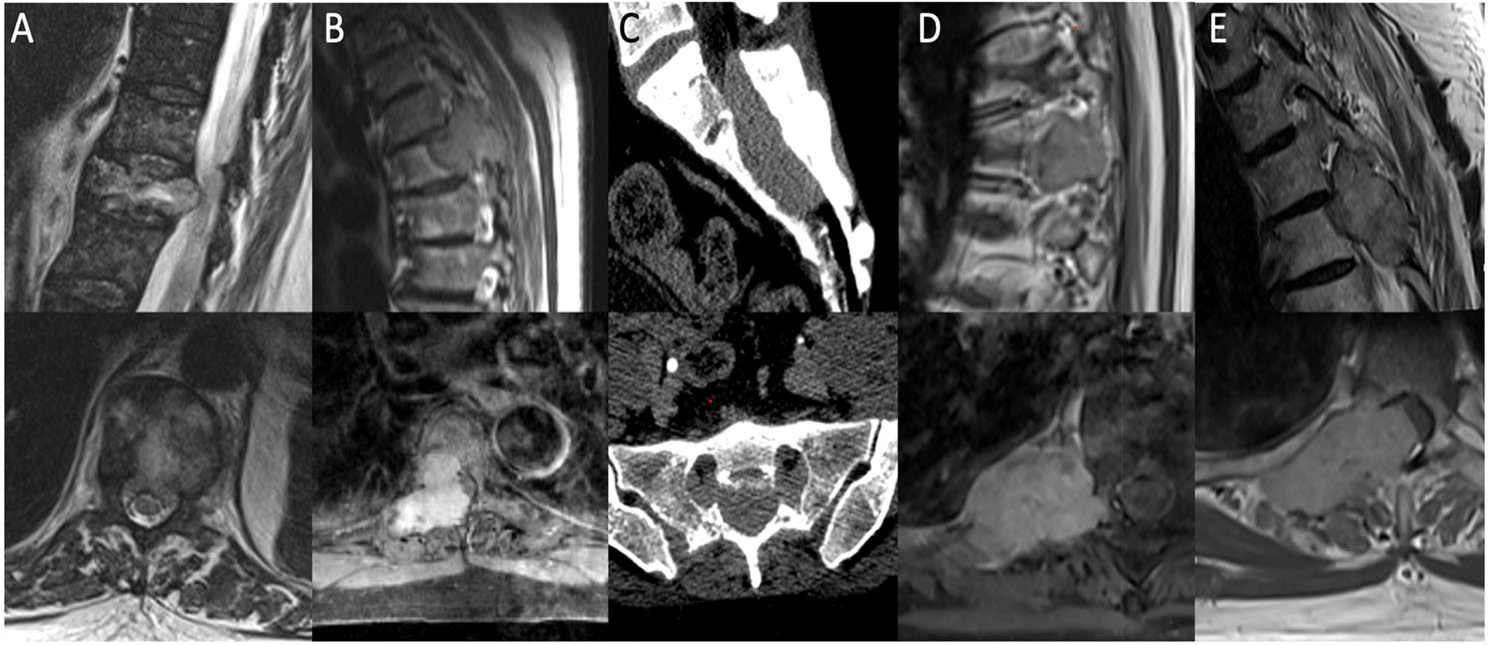
Preoperative imaging of patients #1–#5 (**A**–**E**). For each patient, the upper panel shows a sagittal view and the lower panel an axial view. MRI was performed in all patients except patient #3, who underwent only CT imaging. **A** Patient #1: vertebral metastasis with pathologic fracture of T10 and epidural extension into the spinal canal. **B** Patient #2: mass lesion arising from the right pedicle of T6, extending toward the right ribs and into the spinal canal. **C** Patient #3: predominantly intraspinal mass lesion at S1–S3. **D** Patient #4: predominantly paraspinal mass lesion involving the right pedicle of T7 and the neural foramina without spinal canal involvement. **E** Patient #5: intra- and extraspinal mass lesion involving T3 and T4 on the right side

Contrast-enhanced MRI or CT was available in all cases and demonstrated primarily: #1 a vertebral metastasis with fracture of T10 and spinal canal involvement; #2 a mass lesion arising from the right pedicle of T6, extending to the right ribs and into the spinal canal; #3 a predominantly intraspinal mass lesion at S1–S3; #4 a predominantly paraspinal mass lesion involving the right pedicle of T7 and the neural foramina without spinal canal involvement; and #5 an intra- and extraspinal mass lesion involving T3 and T4 on the right side. Synchronous distant metastases were identified in four patients, involving the lungs (*n* = 4), bones (*n* = 2), liver (*n* = 1), and brain (*n* = 1). The preoperative imaging findings for all five patients are illustrated in Fig. 1.

### Surgical management and adjuvant therapy

Surgeries were performed according to departmental standard techniques (Esmaeilzadeh 2020, Cinibulak 2021). Given the aggressive progression of the metastasis in patient #1, despite repeated therapy, and the instability caused by the T11 fracture, en-bloc resection of T11 with vertebral body replacement and dorsal transpedicular fixation was performed. Patient #2 underwent subtotal tumor resection followed by dorsal transpedicular fixation. In patient #3, subtotal resection and sacral reconstruction with a mesh implant were carried out. The remaining patients underwent subtotal tumor resection only. Patient #1 experienced significant intraoperative blood loss requiring multiple transfusions, whereas all other procedures were uneventful, with minimal blood loss and no intraoperative complications. Histopathological analysis confirmed metastatic FTC in all cases.

Adjuvant treatment modalities included thyroidectomy, radioactive iodine therapy, and radiotherapy. Total thyroidectomy had been performed prior to spinal surgery in 3 patients: in patient #1, 14 years earlier following the diagnosis of TC, and in patients #3 and #4, 16 and 17 years earlier, respectively, due to nodular goiter. Patient #2 had undergone partial thyroidectomy 23 years earlier, due to nodular goiter. In patients #2 and #5, total thyroidectomy was carried out subsequent to spinal surgery and histopathological confirmation. All patients received radioactive iodine therapy after surgery, and patients #2, #4, and #5 additionally underwent radiotherapy.

### Outcomes, follow-up and survival data

The median postoperative KPS was 90% (range: 60–100%), remaining stable in 3 patients and improving in 2 patients. The median follow-up time was 73 months (range: 19–187 months). Patients #3 experienced a recurrence 17 months after initial surgery; there were no other recurrent spinal metastases. Two patients (cases #2 and #3) developed new distant metastases during follow-up, including one spinal metastases and multiple pulmonary and hepatic lesions, despite systemic therapy. At the time of the last follow-up, 4 patients had died and one was still alive. The median overall survival after diagnosis of spinal metastases was 69 months (range: 19–188 months). Treatment and outcome are summarized in Table 2.

**Table 2:** Treatment and outcome of 5 patients with spinal metastases follicular thyroid carcinoma

Case	Operation for thyroid	TNM stage at diagnosis	Co-existing other metastases	Adjuvant therapy	Overall survival after SM diagnosis (months)
#1	Total thyroidectomy	T3 N0 M1	Lung, bone	RAI	188
#2	Total thyroidectomy	Tx Nx M1	Lung, bone	RAI	118
#3	Total thyroidectomy (16 years earlier)	Tx Nx M1	Lung, brain	RAI	19
#4	Total thyroidectomy (17 years earlier)	T3 N1 M1	Lung, liver	RAI	21
#5	Total thyroidectomy	T2 N1 M0	None	RAI	Alive (last follow-up: 73 months)

*RAI* radioactive iodine therapy

## Discussion

In this descriptive case series of five patients with spinal metastases from FTC, surgical management consisted of one en-bloc resection and four subtotal resections, followed by universal radioactive iodine therapy and selective radiotherapy. Neurological and functional status improved postoperatively and the median overall survival was 69 months. Previously published cohorts with spinal metasases from TC are summarized in Table 3.

**Table 3:** Previously published cohorts with spinal metastasis from TC

Author, Year	Number of cases	Histology	Surgical Treatment	Adjuvant / Non-Surgical Treatment
Demura et al., 2011 [14]	24	FTC (*N* = 15) PTC (*N* = 8) MTC (*N* = 1)	Debulking (*N* = 14) TES (*N* = 10)	RAI (not systematically reported)
Quan et al., 2012 [27]	8	FTC (*N* = 3) PTC (*N* = 4) MTC (*N* = 1)	Decompression ± stabilization (*N* = 5) Vertebroplasty (*N* = 3)	RAI (*N* = 8); EBRT (*N* = 1)
Jiang et al., 2014 [15]	21	FTC (*N* = 17) PTC (*N* = 4)	TES (*N* = 3) Decompression ± stabilization (*N* = 18)	RAI (*N* = 14); EBRT (*N* = 6)
Kushchayeva et al., 2014 [6, 21]	37 (institutional cohort) + 165 (literature-derived cases)	FTC (*N* = 120) PTC (*N* = 54) MTC (*N* = 9) HCC (*N* = 6) ATC (*N* = 2)	Procedures not systematically reported (*N* = 122)	Radiosurgery (*N* = 7); radiotherapy (*N* = 87)
Sellin et al., 2015 [16]	43	FTC (*N* = 19) PTC (*N* = 9) MTC (*N* = 6) HCC (*N* = 6) MTC (*N* = 1) PDTC (*N* = 2)	Vertebroplasty (*N* = 24) Vertebral body reconstruction with cage (*N* = 6) Lumbosacropelvic fixation (*N* = 2)	RAI (*N* = 31); EBRT (*N* = 20); SRS (*N* = 3)
Zhang et al., 2019 [17]	52	FTC (*N* = 43) PTC (*N* = 7) MTC (*N* = 2)	En-bloc resection + stabilization (*N* = 8) Curettage/decompression + stabilization (*N* = 44)	RAI (*N* = 52); radiotherapy (*N* = 12); bisphosphonates (*N* = 15); chemotherapy (*N* = 2)
Yin et al., 2021 [28]	50	FTC (*N* = 34) PTC (*N* = 8) HCC (*N* = 8)	Resection ± stabilization (*N* = 16)	Spine radiotherapy (*N* = 28); SRS (*N* = 12); RAI (*N* = 7); systemic therapy (*N* = 32); bone-directed therapy (*N* = 25)
Kleinschmidt-DeMasters et al., 2022 [11]	21	FTC (*N* = 10) PTC (*N* = 3) MTC (*N* = 1) ATC (*N* = 3) PDTC (*N* = 4)	Resection (*N* = 3)	Not reported
Planty-Bonjour et al., 2022 [19]	51	FTC (*N* = 24) PTC (*N* = 22) MTC (*N* = 5)	Decompression ± stabilization (*N* = 10)	EBRT (*N* = 5); RAI (*N* = 31); chemotherapy (*N* = 8); spine radiotherapy (*N* = 21)
Liu et al., 2023 [9]	35	FTC (*N* = 27) PTC (*N* = 8)	TES (*N* = 4) Debulking (*N* = 31)	RAI (*N* = 25); radiotherapy (*N* = 17); zoledronic acid (*N* = 8)
Chaliparambil et al., 2024 [8]	12	FTC (*N* = 6) PTC (*N* = 4) HCC (*N* = 2)	Resection (*N* = 11)	Radiotherapy (*N* = 7)
Ahmed et al., 2024	44	FVPTC (*N* = 34) FTC (*N* = 5) PDTC (*N* = 3) HCC (*N* = 1) - MTC (*N* = 1)	Corporectomy (*N* = 1) Anterior fusion (*N* = 1) Corporectomy + Fusion (*N* = 8) Decompression ± stabilization (*N* = 16)	RAI (*N* = 37); EBRT (*N* = 27)
Matsumoto et al., 2013 [22]	8	FTC (*N* = 6) PTC (*N* = 2)	TES (*N* = 9)	RAI (*N* = 7); preoperative radiotherapy (*N* = 1)

*ATC* Anaplastic thyroid carcinoma, *EBRT* External beam radiotherapy, *FTC* Follicular thyroid carcinoma, *FVPTC* Follicular variant of papillary thyroid carcinoma, *HCC* Hürthle cell carcinoma, *MTC* Medullary thyroid carcinoma, *PDTC* Poorly differentiated thyroid carcinoma, *PTC* Papillary thyroid carcinoma, *RAI* Radioactive iodine therapy, *SRS* Stereotactic radiosurgery, *TES* Total en-bloc spondylectomy

All patients in our cohort were confirmed to have FTC based on histopathologic examination. This pattern is consistent with previously published cohorts reporting a predominance of FTC among spinal metastases from TC (see Table 3) [8, 9, 11, 14–22]. Although PTC accounts for more than 70% of all TC [3, 5], spinal metastases are observed more frequently in FTC. This difference might reflect their distinct patterns of dissemination: FTC characteristically invades blood vessels and spreads hematogenously, predisposing to distant skeletal metastases, whereas PTC predominantly spreads via the lymphatic system to regional cervical lymph nodes [23]. Furthermore, spinal metastases from TC occur mostly in the thoracic vertebrae, as observed in our series, followed by metastases to the lumbar and cervical vertebrae, likely spreading via the valveless Batson’s venous plexus [3]. Also, metastatic spinal cord compression requiring spinal decompression is more frequent in the upper thoracic segments due to the narrower ratio of the spinal canal to the spinal cord at these levels [24].

The decision to consider surgical resection of spinal metastases is typically guided by factors such as neurological symptoms, spinal instability, and the severity of pain, particularly when symptoms significantly affect quality of life or do not respond to conservative treatment [1, 9]. In the emergency setting, surgical intervention is generally aimed at relieving compression of the spinal cord and nerve roots and providing mechanical stabilization. The choice of surgical approach depends on the anatomical location and extent of the lesion, as well as the patient’s overall clinical status [12]. In cases of anterior spinal cord compression, corpectomy or vertebrectomy may be required, while laminectomy is typically performed for posterior decompression. Once decompression is achieved, stabilization of the affected spinal segment is critical to provide structural support and prevent further collapse [25, 26]. Our approach including spinal decompression and stabilization aligns with the predominant surgical philosophy described by other authors (see Table 3) [8, 9, 11, 15, 16, 18, 19, 21, 27, 28]. The use of subtotal resection in the majority of our cases is comparable to the distributions reported by Sellin et al. and Liu et al., where debulking procedures predominated [9, 16]. En-bloc resection was selectively performed in our cohort and, consistent with previously published series [9, 14, 15, 17], was reserved for carefully selected patients with limited spinal disease and adequate performance status. Postoperative functional improvement in our patients, reflected by an increase in median KPS from 70% to 90%, is concordant with functional improvements described by Liu et al. and Yin et al., who reported significant improvement in performance status and quality of life following surgery [9, 28].

The management of the primary TC and of systemic metastases becomes the focus of care after surgical resection and stabilization. Multidisciplinary collaboration involving oncologists, endocrinologists, and radiation oncologists might be essential for further treatment planning [3, 7]. Adjuvant treatment patterns in our series, including universal RAI and selective radiotherapy, mirror the multimodal strategies reported across the literature. In previous studies, RAI was frequently administered in differentiated cases [9, 17, 18, 20, 21, 27]. For adujvant local tumor control, radiotherapy was commonly applied [8, 19, 28]. Similar to larger cohorts, recurrence in our series was uncommon locally but systemic progression occurred in selected patients, consistent with the natural history described by Slook et al. and Planty-Bonjour et al. [19, ]. In contrast to our findings, several investigators have reported the use of additional systemic approaches including bisphosphonates, zoledronic acid or tyrosine kinase inhibitors [9, 17, 19, 20, 28]. The reported median overall survival of 69 months after diagnosis of spinal metastases falls within the range reported by larger cohorts [9, 19].

Our study has several limitations. First, as a retrospective study, it cannot fully ensure subject homogeneity. Second, the sample size is small. Finally, the study is based on cases from a single institution rather than multiple centers, so the findings should be interpreted with caution.

## Conclusion

In this descriptive case series, surgical management of spinal metastases from TC was performed primarily to address neurological compromise and spinal instability. The operative objective was preservation of neurological function and structural stabilization, followed by comprehensive evaluation and management of the primary malignancy. Multidisciplinary coordination remains central to patient care, with subsequent treatment directed toward control of systemic disease.

## Data Availability

Data available from the corresponding author upon reasonable request.
